# Application of unsupervised analysis techniques to lung cancer patient data

**DOI:** 10.1371/journal.pone.0184370

**Published:** 2017-09-14

**Authors:** Chip M. Lynch, Victor H. van Berkel, Hermann B. Frieboes

**Affiliations:** 1 Department of Computer Engineering and Computer Science, University of Louisville, Louisville, KY, United States of America; 2 Department of Cardiovascular and Thoracic Surgery, University of Louisville, Louisville, KY, United States of America; 3 Department of Bioengineering, University of Louisville, Louisville, KY, United States of America; 4 James Graham Brown Cancer Center, University of Louisville, Louisville, KY, United States of America; Harbin Institute of Technology Shenzhen Graduate School, CHINA

## Abstract

This study applies unsupervised machine learning techniques for classification and clustering to a collection of descriptive variables from 10,442 lung cancer patient records in the Surveillance, Epidemiology, and End Results (SEER) program database. The goal is to automatically classify lung cancer patients into groups based on clinically measurable disease-specific variables in order to estimate survival. Variables selected as inputs for machine learning include Number of Primaries, Age, Grade, Tumor Size, Stage, and TNM, which are numeric or can readily be converted to numeric type. Minimal up-front processing of the data enables exploring the out-of-the-box capabilities of established unsupervised learning techniques, with little human intervention through the entire process. The output of the techniques is used to predict survival time, with the efficacy of the prediction representing a proxy for the usefulness of the classification. A basic single variable linear regression against each unsupervised output is applied, and the associated Root Mean Squared Error (RMSE) value is calculated as a metric to compare between the outputs. The results show that self-ordering maps exhibit the best performance, while k-Means performs the best of the simpler classification techniques. Predicting against the full data set, it is found that their respective RMSE values (15.591 for self-ordering maps and 16.193 for k-Means) are comparable to supervised regression techniques, such as Gradient Boosting Machine (RMSE of 15.048). We conclude that unsupervised data analysis techniques may be of use to classify patients by defining the classes as effective proxies for survival prediction.

## Introduction

The ability to estimate lung cancer survival based on disease attributes from past patient populations may be of value when treating particular patients, and could thus complement current clinical practice. With the goal to minimize necessary expertise required for such database analysis while also providing for the opportunity to obtain novel insight, in this study unsupervised learning techniques are evaluated to automatically analyze lung cancer data available from the Surveillance, Epidemiology, and End Results (SEER) program of the National Cancer Institute (NCI) [1, 2]. The SEER Program is an authoritative repository of cancer statistics in the United States [3]. The SEER database enables outcome analysis for large number of patients based on attributes broadly classified as diagnostic (e.g., surgical procedure, radiation therapy), demographic (e.g., age, gender, location), and outcome (e.g., survival time, cause of death). Further detailed information about the lung cancer dataset can be found at the website of the NCI [4].

Past work has analyzed patient survival from the SEER database based on different attributes for various cancers. These attributes have included age [5, 6], number of primaries [7, 8], smoking status [9], and gender [10]. A comparative analysis of lung cancer incidence rates in the U.S. was performed in [11]. Additional work has evaluated survival rates for rectal [12] and limited stage small cell cancer [13]. Prediction models for survival time or a number of other factors have been explored; typically, these efforts have involved supervised machine learning classification techniques, data mining and statistics [14–25]. In terms of machine learning, supervised learning algorithms categorize records based on labeled data. The process involves collecting and labeling a particular dataset, and then developing or customizing correlation techniques for the dataset. The functions inferred from the labeled training data can then be used to classify new data. In contrast, unsupervised techniques do not use labeled data; the process is based on measuring the similarity of “intra” classes and dissimilarity of “inter” instances while minimizing *a priori* assumptions. For example, cluster analysis uses unlabeled input data to create groupings that may facilitate data analysis. Of note, semi supervised techniques use a small group of labeled data, with the model updated as new data is added to the set. The application of any one technique may be complicated by factors such as incomplete (missing) patient data, which can affect the quality of survival prediction [26, 27].

Application of supervised methods requires a certain level of technical expertise. Common techniques include Decision Trees, Gradient Boosting Machine, and Support Vector Machines. Decision Trees decomposes a dataset into smaller subsets while creating a decision tree associated with these data; the final designation of the subset is decided at a single leaf or end node where the data subset cannot be further split. In particular, the Random Forest technique creates a number of decision trees during training which split randomly from a seed point. This process yields a “forest” of randomly generated decision trees whose outcomes are integrated as an “ensemble” by the algorithm to predict more accurately than a single tree would. In comparison, Gradient Boosting Machine (GBM) uses weaker, smaller models to create an “ensemble” to produce a final prediction. New weak models are iteratively trained with respect to the current whole ensemble. The new models are built to be maximally correlated with the negative gradient of the loss function that is also associated with the ensemble as a whole. In contrast, Support Vector Machines (SVM) is an example of non-probabilistic binary linear regression. Given a set of training data labeled as belonging to one of two sets, the technique represents the sets in space and defines a hyper-plane separating them that is maximally distant from both sets. If a linear separation is impossible, the technique applies kernel methods to perform non-linear mapping to a feature space, in which the hyper-plane represents a non-linear decision boundary in the input space.

In recent years, supervised, semi-supervised, and unsupervised machine learning techniques have found wide application to help analyze genomic, proteomic, and other types of biological data [28–31], with Random Forest (e.g., [32–34]) and SVM (e.g., [35–37]) playing major roles. Here, we explore the capability of unsupervised machine learning techniques for lung cancer patient survival prediction. These techniques inherently involve less human expertise and interaction than supervised methods, and thus minimize required intervention for database analysis. A number of unsupervised techniques is applied to evaluate their performance in clustering patients with similar attributes. Although unsupervised techniques have been previously applied to evaluate breast cancer patient survival [38, 39], to the best of our knowledge this work represents the first time such approaches have been evaluated with respect to lung cancer data. Longer term, the automated classification of patients into groups may facilitate comparison and evaluation of prognostic as well as diagnostic considerations in clinical practice.

## Materials and methods

### Disease variables

The SEER database contains hundreds of variables, with more than 30 of them meaningful to the Lung Cancer patient group. Clustering, by its very nature, is designed to minimize the distance between similar data points–patients in this case–and maximize the distance between dissimilar groups of points. For the purposes of this study, only variables that can be represented numerically were selected as inputs for machine learning. These variables include Number of Primaries (1–7), Age (22–87, in groups of 5), Grade (1–4), Tumor Size (0 to 989mm), Stage (1–4), T (0–4), N (0–3), and M (0–1). Non-numeric information such as the types of procedures performed (“Beam Radiation”, Intra-“operative Radiation”, etc.) were excluded from classification since some machine learning techniques do not directly support non-numeric inputs. There are options–such as one-hot encoding of “dummy” variables–to facilitate numeric analysis of categorical data, but they were excluded here due to the sparsity of populated data in these categories in our dataset and to minimize the pre-processing effort in what is designed to be a black-box algorithm comparison.

The SEER Data Dictionary describes the selected variables as follows.

Age: Three-digit code that represents the patient’s actual age in years. (Here, a single 999 record was removed.)

Grade: Grading and differentiation codes 1 through 4 are defined in ICD-O-2; 1992 [2]. Grade represents cell appearance under microscopic examination, and is usually obtained from biopsied tissue. Non-small cell lung cancer can be classified into four grades: Grade 1, for which cells may look normal but with some evidence of proliferation; Grade 2, for which cells look abnormal but growth seems restrained; Grade 3, for which cells look poorly differentiated with clear evidence of growth; Grade 4, for which cells look poorly differentiated and with evidence of strong growth.

Tumor Size: Records the tumor size in mm. Codes 991–995 indicate “Described as less than x cm” where x is the last digit (1 through 5). The codes were converted for these ranges to median tumor sizes (cm).

T, N, and M: These are the American Joint Committee on Cancer (AJCC) “T”, “N”, and “M” components that are derived from Collaborative Stage (CS) coded fields, representing “tumor,” “node,” and “metastasis,” respectively, using the CS algorithm, effective with 2004+ diagnosis. See the CS site-specific schema for details [40]. The meaning of T, N, and M values is described in **Table 1** [41].

**Table 1:** 

**“T” (Tumor)**	
T0	No evidence of a primary tumor
T1	Tumor is < 3 cm in diameter, has not penetrated the visceral pleura, and has not affected the main bronchi branches. If tumor is < 2 cm, it is T1a stage; otherwise, it is T1b.
T2	Tumor is > 3 cm in diameter, or has penetrated the visceral pleura, or has partially occluded the airways, or involves the main bronchus but is > 2 cm away from the carina. If tumor is < 5 cm, it is T2a stage; otherwise, it is T2b.
T3	Tumor is > 7 cm in diameter, or has caused an entire lung to collapse or develop pneumonia, or has grown into the chest wall, diaphragm, mediastinal pleura, or parietal pericardium, or involves the main bronchus and is < 2 cm from the carina (without involving the carina), or two or more tumor nodules are present in the same lung lobe.
T4	Tumor has grown into the mediastinum, heart, large blood vessels near the heart, trachea, esophagus, spinal column, or the carina.
**“N” (Node)**	
N0	No tumor spread to nearby lymph nodes
N1	Tumor has spread to lymph nodes within the lung or near the hilar lymph nodes. The affected lymph nodes are on the same side of the body as the primary tumor.
N2	Tumor has spread to lymph nodes around the carina or in the mediastinum. Affected lymph nodes are on same side as primary tumor.
N3	Tumor has spread to lymph nodes near the clavicle on either side, or spread to hilar or mediastinal lymph nodes on opposite body side of primary tumor.
**“M” Metastasis**	
M0	Tumor has not spread to distant organs or to the other lung or lymph nodes farther away than in those specified in the “N” classification.
M1	Tumor has spread to distant organs or to the other lung or lymph nodes farther away than in those specified in the “N” category.

Definition of T, N, and M categories for Non-Small Cell Lung Cancer [41].

Stage: This is the AJCC “Stage Group” component that is derived from CS coded fields, using the CS algorithm, effective with 2010+ diagnosis. See the CS site-specific schema for details [40]. The information from the T, N, and M components is combined to define the Stage value, as described in **Table 2** [41].

**Table 2:** 

Stage	TNM Category		
Grouping	T	N	M
1A	T1	N0	M0
1B	T2a	N0	M0
2A	T1	N1	M0
	T2a	N1	M0
	T2b	N0	M0
2B	T2b	N1	M0
	T3	N0	M0
3A	T1 to T3	N2	M0
	T3	N1	M0
	T4	N0 or N1	M0
3B	T1 to T4	N3	M0
	T4	N2	M0
4	T1 to T4	N1 to N3	M1

Stage grouping for Non-Small Cell Lung Cancer based on TNM category [41].

### Classifying techniques

The unsupervised techniques evaluated in this study are common, widely available (particularly through open source implementations), well researched and supported, and representative of available approaches.

Hierarchical Clustering–Relatively simple and established technique to iteratively divide a data set into a tree of increasingly smaller clusters. The approach takes into account the Euclidean distance between the scaled input variables and iteratively breaks the single data set into more and more clusters as individual distance thresholds are crossed. The result is a tree structure where a “cut” can be made at any height resulting in a number of clusters between 1 and the number of records.

Model-Based Clustering (MBC)–Complex technique focused on Expectation Maximization (EM) through normal mixture models and cutoff estimation based on the Bayesian Information Criteria (BIC), which attempts to select not only a particular underlying model, but an optimal number of clusters without any extra guidance [42, 43].

k-Means Clustering–A common, simple classification technique which requires users to specify the number of classes ahead of time. The technique, while unsupervised in the basic sense, has no built-in mechanism for determining the optimal number of clusters, thus requiring the number of clusters k to be provided manually. Unlike hierarchical clustering, for which a cutoff needs to be supplied after building the hierarchy to establish classes, k-Means requires the input k variable at the outset.

Self-Ordering (Kohonen) Maps–Self-ordering maps (SOMs) project high dimensional data into lower dimensional (typically 2D) clusters [44]. SOMs apply the idea of splitting data into classes, but rather than a single number of classes, the technique divides the data into a map; essentially a 2-D grid of squares or hexagons that each represent an individual class. Because of this approach, SOMs are often used to classify data into larger numbers of classes; typically evenly sized grids of 5x5, 10x10, 20x20, or higher maps, resulting in tens or hundreds of classes. Unlike other classification techniques, these classes are related by their proximity on the map. In other models there is no reason to believe that any pair of classes would be more or less similar than others. Although the high number of classes is more difficult to manipulate when manually comparing between classes and across models, SOMs lend themselves well to data visualization using the grids as actual visual maps.

### Non-classifying techniques

Some unsupervised techniques do not separate the dataset into classes directly; rather, they build feature sets (variables) that attempt to condense the information in high density data sets into a much smaller number of dimensions. The output can sometimes be converted to or interpreted as classifications, but it is not necessarily considered a distinct goal. It has been shown [45–49] that data with a large number of dimensions can cause over-fitting leading to low generalizability of a model, information redundancy or noise, all of which can result in low model accuracy and inefficiency. In this study the following techniques were evaluated to explore whether reducing the system dimensions would optimize the feature sets.

Non-Negative Matrix Factorization–In NMF, a data set (X) is deconstructed into two matrixes W and H such that *X* ≈ *W* × *H* where W and H are typically much lower dimension matrixes than X [50]. Thus, NMF attempts to decompose input data into two matrixes which, when multiplied, estimate the original data. This is not, strictly speaking, classification, however it is common to treat the rank of one of the matrices as a number of classes, and reverse engineer the matrix multiplication to assign each record to one of those ranks based on which rank contributes most to the record’s values. Like k-Means or hierarchical clustering, this rank–r–must be supplied. NMF algorithms typically provide some mechanisms for estimating reasonable values of r.

Principal Component Analysis–PCA builds a set of *n* highly orthogonal variables PCA1, 2, …, *n* that represent the original data set by reducing the correlation between any two variables; in theory there will be fewer of these principal components than there are original variables in a real-life data set with interdependencies. PCA thus decomposes the input data into variables in such a way as to reduce correlation between those variables; making them in theory more independent, and reducing the number of variables in the process. The method does not provide a direct way to classify individual records, although it still provides a means for breaking down the variables into a smaller number of less highly correlated variables.

### Code implementation

The language R was used for implementation, as it is an open source statistical programming platform with access to machine learning algorithms. In order to maintain the “black box” benefit of unsupervised methods intact, the parameters for the various chosen methods were mostly kept to default values. When choices had to be made, the parameter selection is explained in the individual model section (Results), while parameters such as the number of bins were chosen to enable comparison across models. For instance, the Model-Based Clustering automatically selected 9 buckets as optimal, so k-Means was also tested with 9 classes.

To ensure generally comparable results, a small amount of pre-processing (such as scaling and centering the variables to be of comparable values in the distance measures used in machine learning) was performed and included in the R code [51].

### Number of records

A total number of 10,442 records from the SEER database for patients diagnosed with lung cancer between the years 2004–2009 were in the chosen dataset for unsupervised classification analysis.

## Results

### Hierarchical clustering

**Fig 1:**
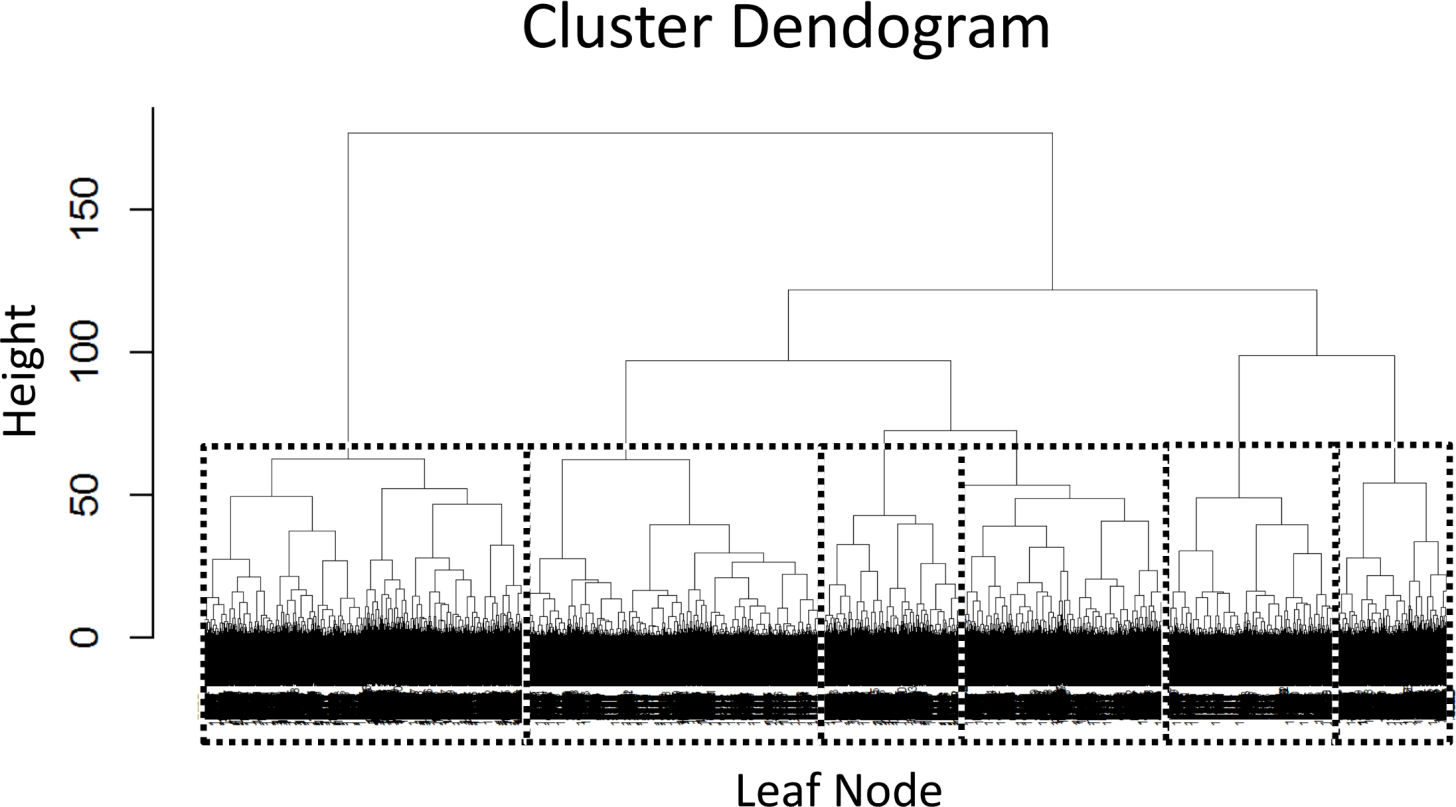
Hierarchical clustering dendrogram with class selection.

Applying Hierarchical Clustering to the SEER Lung Cancer data yields a hierarchy of cluster separations which can be visualized as the dendrogram–a common representation of these tree structures (**Fig 1**). In the figure, a dashed box is added that divides the data into six clusters, selected by moving down the chart until a point is found where the horizontal line crosses exactly six branches in the dendrogram. With over 10,000 records, the visualization becomes very dense at the bottom of the tree, although for the purposes here there is no need to distinguish between the leaves.

What Hierarchical Clustering fails to do is provide an idea of where one should draw the line splitting the resulting clusters. It simply generates every successive classification descending into the tree until every record is individually separated. If one had a number of classes in mind, such as the 6 chosen in **Fig 1**, this would be a useful approach, but more information is required to generate a useful collection in a completely unsupervised fashion.

### Model-based clustering

Running a Model-Based Cluster (MBC) with no additional tuning results in a set of 9 clusters, with the distribution of records as in **Table 3**.

**Table 3:** 

**Technique**	Class 1	Class 2	Class 3	Class 4	Class 5	Class 6	Class 7	Class 8	Class 9
**Model-Based**	1588	1314	637	835	1071	727	774	939	2557
**k-Means**	992	1098	1154	1612	1146	651	1232	926	1631

Distribution of records based on applying the Model-Based and k-Means clustering techniques.

### k-means clustering

With k = 9, as an example selected manually to match the automatic number of classes from the MBC, a different distribution of classes is obtained (**Table 3**). Interestingly, the classes do not comprise the same members for both models. In general, the purpose of using multiple unsupervised clustering techniques is to be able to compare results between them. For example, k-Means can be compared to the previous MBC, obtaining the distribution of classes as shown in **Table 4**. This mostly serves as a check that the models agree in some facets while differing in others. The k-Means clusters 3 and 7 are associated with MBC clusters 1 and 5 respectively, while k-Means cluster 4 is predominately associated with MBC clusters 4 and 7, while MBC cluster 9 is split between k-Means clusters 1 and 9. The order of the clusters is essentially random, which is why agreement does not follow the diagonal as may be expected.

**Table 4:** 

Model-Based	k-Means								
	1	2	3	4	5	6	7	8	9
**1**	0	36	1094	28	0	429	1	0	0
**2**	21	95	0	63	17	214	44	860	0
**3**	26	101	21	61	117	8	240	18	45
**4**	0	87	0	675	52	0	9	12	0
**5**	0	18	39	15	52	0	938	9	0
**6**	0	727	0	0	0	0	0	0	0
**7**	0	0	0	754	20	0	0	0	0
**8**	0	12	0	16	888	0	0	23	0
**9**	945	22	0	0	0	0	0	4	1586

Comparison of k-Means to the Model-Based Clustering. While there are some areas where hundreds of members are modeled into the same class, few member counts dominate both the Model-Based and k-Means based classification at the same time, implying that there is some, but not too much, agreement between the two methods.

### Visualizing cluster results

**Fig 2:**
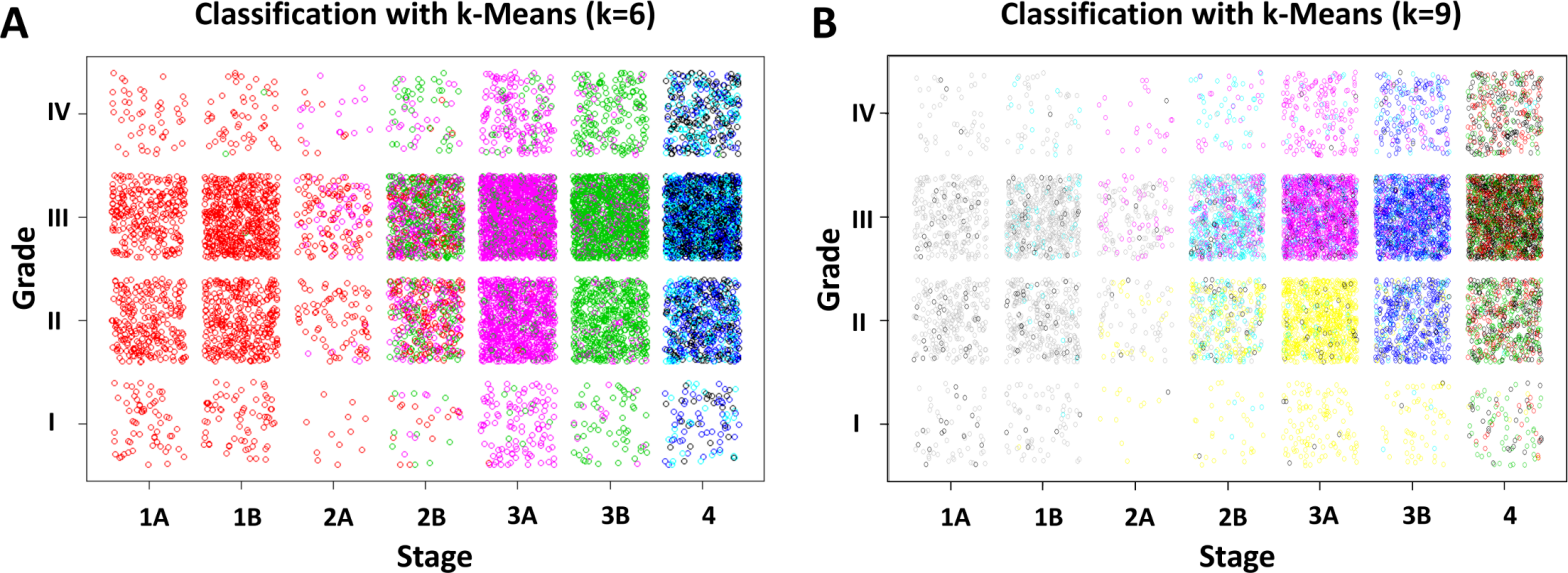
Colored classification exhibited by cancer Stage and Grade using k-Means algorithm. (A) Classification with k = 6. (B) Classification with k = 9. Some jitter was added to allow the individual points to be more discernable within boxed regions.

With even just a few variables, visualizing clusters becomes difficult. One solution is to examine any two variables at a time and examine the class correlation. The cancer Stage and Grade variables (converted to numbers), yield the classifications shown in **Fig 2**.

**Fig 2A** presents the Stage (x-axis) and Grade (y-axis) classified with k-Means with k = 6. Stages 1A, 1B and 2A are dominantly in a single class regardless of grade, while Stage 2B seems to have a variety of pink and green classes, which are more segregated in Stages 3A and 3B, while Stage 4 is dominantly blue. In contrast, the grades appear evenly distributed in terms of color. Note that scaled proxy values were used in the models and not the categorical stage indications of 1A, 1B, etc. The clinically recognizable values were used where not directly referencing the model inputs in order to make the charts more readily interpretable. These results show that the k-Means algorithm with six classes was able to separate patients with cancers in different stage, while grade was not necessarily a distinguishing characteristic between these classes.

In **Fig 2B**, the k-Means algorithm with 9 classes was able to separate grade as well as stage into multiple classes for certain combinations. The yellow and pink classes (colors are not comparable between panels (**A**) and (**B**); they simply represent groupings within a chart), are split between Grades I/II and III/IV, with Grades I/II having more yellow while Grades III/IV are dominantly light blue or pink for stages 2B and 3A, respectively. In comparison, stage 3B splits grades differently, with Grade 1 being mostly yellow, while Grades 2, 3, and 4 are darker blue.

**Fig 3:**
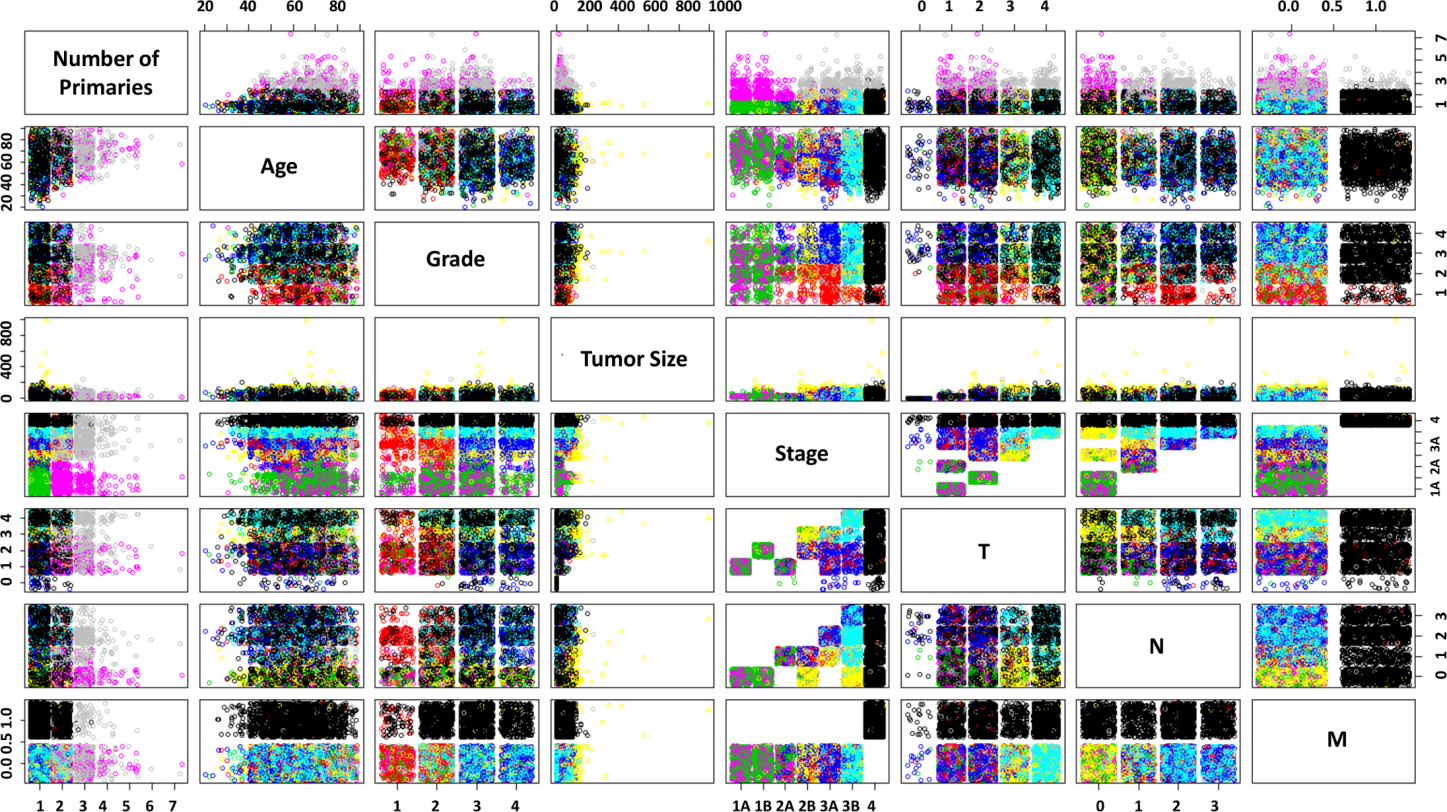
Pairwise plot of classes for all variables for k-Means with 9 groups.

Every possible pair of classes is plotted on a single chart in **Fig 3**, colored with the groups from the k-Means with 9-classes. The TNM staging components are of interest, for example: M values are split such that M = M1 are almost entirely in one class and the remaining classes are in M0. N and T share some classes; N = N0 and N1 contain the yellow class, which is strongest in T = T3, and these are common with large Tumor Sizes. This is what the T factor indicates; interestingly, it is T3 and not T4 that is most strongly associated with large actual Tumor Sizes for the yellow class, so one cannot deduce that yellow is solely a proxy for large tumor sizes; it is also associated with N = 0 and stages 2B, 3A, and 3B. The class colored green is dominant in records with Stage 1–2 and a low number of primaries. The red clusters are associated with low grades, and medium-high stages. These class insights can lead to further detailed analysis, and it is noted how quickly such analysis could be performed with little actual data preparation or up-front intervention. Note that the original class labels were applied rather than the scaled values, while the underlying models were built with the described pre-processed numerical values.

### Self-ordering maps

**Fig 4:**
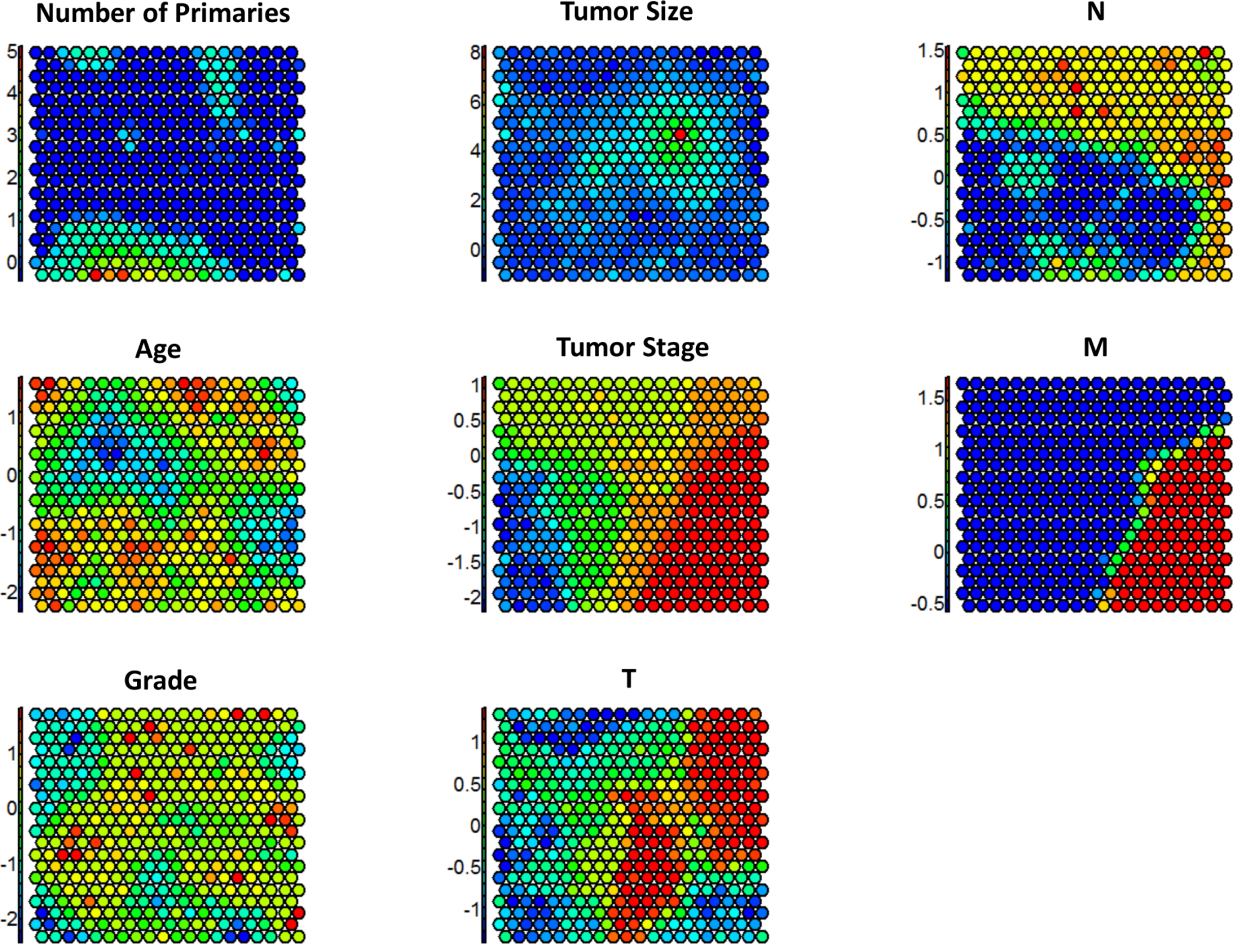
Feature specific class assignments for self-ordering maps.

Seeding a 20x20 map, which results in 400 classes, yields a result which is not as easy to interpret or compare as with other mechanisms, but it does allow for visualization of the distribution of the data. **Fig 4** helps to describe how variables relate to each individual grid point on the map. Although the models were run on scaled data, the charts are plotted against the original, unscaled data in order to visualize the distribution of classes against these clinical data.

Both Tumor Size and Number of Primary sites have strong outliers–points that are more than a few standard deviations from their mean. This makes the maps of these variables less diverse; tumor size in particular shows a fairly even distribution except for one small set of grid points that house the highest tumor sizes. The M field, on the other hand is binary, and it is distinctly spread between the grid points. Points with M = 1 are in red while points with M = 0 are in blue. It is also allowable in these maps to correlate between maps, i.e., data elements in red classes in the Stage map correlate highly to classes with red in the M map, which is visually observable. It is also apparent that Age and Grade are more evenly distributed across the classes.

### Non-negative matrix factorization

**Fig 5:**
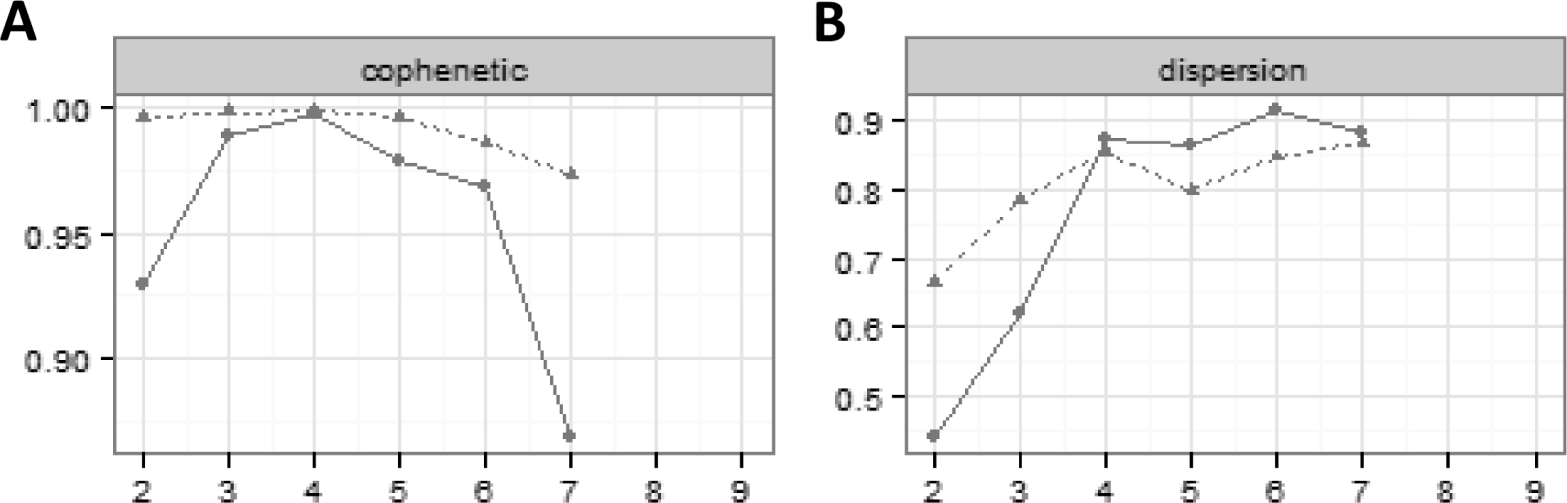
Default output from Non-Negative Matrix Factorization Package for rank selection. Solid lines: original version of the data; dashed lines: randomized version of the data.

The charts in **Fig 5** are created by running NMF with ranks ranging between 2 and 9 for both the original and a randomized version of the data. **Fig 5A** seems to indicate that after 4 ranks the random data and the original data diverge, indicating that there may be some overfitting. The algorithm would not calculate for more than 7 ranks, so this value was the maximum that could be considered. **Fig 5B** clearly peaked and plateaued after 4 ranks. It thus seems that 4 or 5 ranks (or classes) is likely optimal for this algorithm. Selecting r = 5, an evenly distributed set of classes can be built as shown in **Table 5**.

**Table 5:** 

Class 1	Class 2	Class 3	Class 4	Class 5
3610	2787	2197	410	1438

Set of classes built from Non-Negative Matrix Factorization (r = 5).

### Principal component analysis

**Fig 6:**
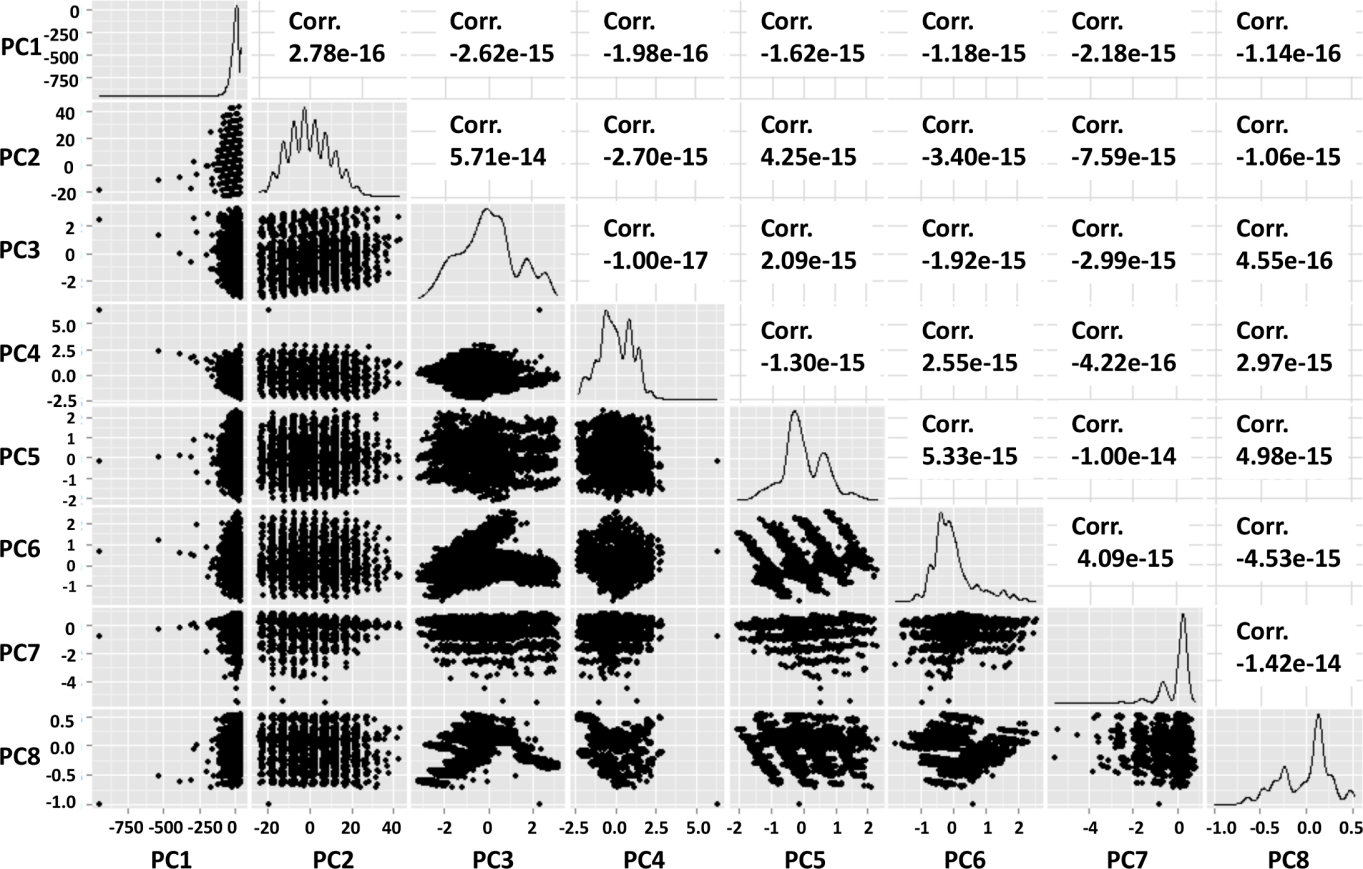
Pairwise scatterplots of PCA decomposition components.

When a naïve PCA analysis is performed on the dataset, a set of 8 variables is created with low pairwise correlation which in principle maintains a significant amount of knowledge of the original data (**Fig 6**). The language R includes a map designed for PCA which shows the relationship between the output principal components and their relative correlation statistics. This is valuable in determining that the PCA is not over-trained; i.e., the result includes only highly orthogonal result vectors, even though there is not necessarily a particular meaning to the original data set of the individual values as there was with the previous classification methods.

Although the pairwise correlations with PCA remain low, certain patterns emerge. PCA2, for example, seems particularly discrete with what appear to be nine discrete groupings (in column two of the chart). The intersection of PC3 and PC6 has a separation into what appear to be two merging clusters, and some other clusters such as PC5 and PC7 also have discretization in four and six sets respectively. Interestingly, the PC1 is skewed to one side, which can be attributed to the same issue that made Tumor Size an outlying feature in the Self-Ordering Map and k-Means charts; namely, that Tumor Size is a skewed metric.

### Comparison of the models

Metrics exist to compare the distance within and between clusters, but having the best fit does not necessarily make a model the most useful. The primary outcome available in the SEER data is Survival Time, so the output of the unsupervised techniques is used to predict survival time, with the efficacy of the prediction representing a proxy for the usefulness of the classification. In practical application, once the utility of unsupervised classification has been established, it can be applied without this step, and a supervised analysis could be used to ensure the meaningfulness of the results. Here, a basic single variable linear regression against each unsupervised output (i.e., survival time vs. the output of the classification) is applied, and the associated Root Mean Squared Error (RMSE) is calculated as a metric to compare the results. This is analogous to assessing patients based solely on the results of the unsupervised classification. Predicting against the full data set and measuring RMSE against this naïve linear regression, the results are obtained as in **Table 6**.

**Table 6:** 

Classification Technique	Root Mean Squared Error of Linear Regression	Coefficient of Determination (R^2^)
Hierarchical Clustering	16.202	0.06819
Model-Based Classification	16.250	0.05659
k-Means Classification	16.193	0.06731
Self-Ordering Maps	15.591	0.13539
Non-Negative Matrix Factorization	16.589	0.01923
Principal Component Analysis (PCA)	16.085	0.07969

Root mean squared error of linear regression and coefficient of determination values for the various classification techniques evaluated. While PCA did not have specific classes, regression against the component values themselves can be performed, which makes the comparison possible albeit slightly less meaningful.

The Self-Ordering Maps shows the best performance with an RMSE of 15.591, while the k-Means (RMSE of 16.193) has the best performance of the simple classification techniques. In comparison, the RMSE of Gradient Boosting Machine is 15.048, which is better than all of these unsupervised results, but is the outcome of a significantly more complex analysis using a fully supervised technique. The coefficient of determination (R^2^) value **v**aries as expected with RMSE (lower RMSE correlates almost directly with higher R^2^). Although these results cannot be compared to those of typical regressions, here the regression has been effectively performed on a single variable (the resulting class) which can assume only a small number of values (the number of classes in the classification itself) as a proxy to compare the classifications. Thus, while unsupervised classification can distill information into a single class, such classifications may have meaningful (RMSE comparable to other methods) but limited (low R^2^) predictive power by themselves.

PCA has the second-best performance overall, but it is difficult to compare to other unsupervised techniques which perform classification; PCA is really performing decomposition into components with no direct clinical meaning. The first component is effectively a mathematical attempt to describe the data in each record for all variables in a single number; the second component adds a second dimension to the decomposition and so on, but these dimensions relate only mathematically to the data, not in a readily-understandable way that can be exploited for clinical use. For this reason the components cannot be treated as classes either; all the records should have most of their information distilled in the first components, and only outliers should have strong values in higher PCA components. Nevertheless, PCA is a common unsupervised method that can be applied, and the results may be of use in comparative evaluations.

## Discussion

This study evaluated unsupervised machine learning techniques to automatically classify lung cancer patients into groups based on disease specific variables in order to estimate survival with minimal human intervention. While models were run numerous times with varying random seeds, they were not tuned in such a way that any particular output was optimized, as is often done with supervised models and models used for specific predictive purposes. The models were intentionally blind to the final output of survivability, which was only used at the end to evaluate whether the classification was informative. Also, the most typical parameter selections and options, such as distance functions and evaluation metrics, were selected to avoid reliance on manual tuning. The only exception was in the selection of the number of classes in some of the models (e.g., as with k-Means). Further, other than a basic scaling and centering option to ensure that models sensitive to skewed distance measures were not overly impacted, there was no significant pre-processing. The purpose of unsupervised classification is to avoid human work (supervision); the inputs used here are already clinically measured and represent straightforward parameters. Ultimately, exploration of these models could be useful if deployed in situations where data is available and quick, unsupervised, “black-box” (where the definitions of the classes are not directly apparent) analysis is practical.

Minimal manual processing was applied in advance of model training in order to ensure the unsupervised learning techniques were exposed to data that represented patient clinical information. As such, records with missing values were largely kept as-is, and classes were not manually balanced, which is considered a better reflection of clinical data. Previous work has sought to address such issues [26, 27]. This study did not attempt to achieve an explicit statistical result, where such considerations would have impact, but instead sought to assess how unsupervised methods could behave on real, untidy clinical data.

Unsupervised techniques may enable classification of patients into groups in an automated fashion, which would allow for caring for patients in the same group in a less subjective manner. This has limitations, however; while computer models are decisive, their output may not make immediate sense, and may lack the empathy or flexibility that a clinician can provide. Still, these analyses remain a potential tool that may reduce complexity. For example, assume that a patient is evaluated for lung cancer; TNM staging is assigned, grade and stage are recorded. Both patient and doctor may be concerned with how the diagnosis compares to other patients in the same category. The unsupervised classifications may be able to indicate more quantifiably which cases are most similar, and what worked or did not work for their treatment. Suppose the patient has T3, N0, M0 values for TNM categorization, thus being classified as a Stage 2B, and the associated histological grade is 3. From **Fig 2B**, one can see that Stage 2B/Grade 3 is mostly teal with some pink, green, black and blue, while Stage 2B/Grade 2 has both teal and yellow with some black and Stage 2B/Grade 4 is teal and pink. Seeing that the teal is a shared classification between these grades, one could assess how this patient group has performed, and what options would be common to the other groups. One may also be able to more easily determine if the patient is an outlier in other areas, e.g., to ascertain whether a particular combination of TNM was rarely seen in patients classified with a low stage or more than two primary sites. This information could later be confirmed, and if not confirmed, it could lead to a re-classification and a change in treatment.

As recently shown in demonstrating new prediction/classification methods (e.g., [52–57]), user friendly and publicly accessible web servers offer the potential to significantly enhance the impact of studies applying machine learning techniques [58]. Accordingly, future work will evaluate providing a web server for the methodology reported herein. Limitations of this study include the restriction of including variables that are numeric or that could be easily converted to numeric; a broader set of both numeric and categorical variables needs to be evaluated in terms of their role in the prediction of survival analysis. Other unsupervised learning techniques such as deep learning and neural networks may be of value in this analysis and should be further explored. The pairwise analysis of classes could be extended to elucidate in more detail relationships between the clinically-relevant variables which determine patient outcomes. Additional variables than in the set chosen here may be evaluated for how they affect the analyses. Inclusion of more detailed patient datasets, including of the–omic categories, may additionally enable translation of these analyses to personalized clinical application.
